# Factors associated with the burden of family caregivers of elderly patients with femoral neck fracture: a cross-sectional study

**DOI:** 10.1186/s13018-020-01749-9

**Published:** 2020-06-23

**Authors:** Peifen Xiao, Yongchun Zhou

**Affiliations:** Department of Orthopedics, Shaanxi Provincial People’s Hospital, Xi’an, Shaanxi People’s Republic of China

**Keywords:** Femoral neck fracture, Caregivers, Elderly, Burden, Factors, Hemiarthroplasty

## Abstract

**Background:**

This study aimed to study the factors associated with caregiver burden among caregivers of elderly patients with femoral neck fracture.

**Methods:**

This cross-sectional study was based on a non-probabilistic sampling of 183 elderly postoperative patients (aged 65 years or older) with femoral neck fracture who were hospitalized in the orthopedic center in our hospital and their family caregivers. Data were collected from January 2016 to June 2019. Patients and family caregivers completed the sociodemographic questionnaire. The Social Support Rating Scale (SSRS), the General Self-Efficacy Scale (GSE), and the Chinese version of the Zarit Burden Interview (ZBI) were used to evaluate social support, self-efficacy, and caregiver burden, respectively. By analyzing the clinical data of patients and family caregivers and combining the factors that affect the caregiver burden in parallel studies, we selected the factors that affected the caregiver burden in this study and conducted a multivariate analysis of these factors. *P* < 0.05 was considered statistically significant.

**Results:**

We observed 176 caregivers aged 69.28 ± 7.19 years old, among whom 52.3% were male, 58.0% lived in the city, 84.0% were spouses of the patients, and 67.0% had a primary school educational background. The ZBI score of the family caregivers was 37.8 ± 8.9, and 82.7% of the caregivers were under a moderate to severe burden. The patient’s functional status, Harris score, and pain score and the caregiver’s SSRS scores, GSE scores, and the ratio of medical expenses to monthly income per capita were factors that affected the caregiver burden.

**Conclusions:**

Most family caregivers of elderly patients with femoral neck fracture are subject to a considerable care burden, and social support and self-efficacy intervention are conducive to reducing the caregiver burden.

## Background

Femoral neck fracture is an injury of the femoral neck that comes under an external force due to either osteoporosis or hip joint muscle decline [1]. Osteoporosis patients are increasingly common in the elderly population and present a high-risk group of femoral neck fractures. With an increasing trend of an ageing population in our country, the incidence of femoral neck fracture has increased each year and has become a major disease contributing to a high disability rate, high fatality rate, and high medical costs, thereby seriously affecting the physical and mental health of the elderly [2, 3]. Hemiarthroplasty (HA) is a common method for treating femoral neck fracture. HA can significantly improve hip joint function, improve quality of life, and reduce complications [4, 5]. Rehabilitation management of patients with femoral neck fracture remains a complex and perennial health issue, and nursing for patients with femoral neck fracture calls for long-term caregiver support.

In most areas in China, due to the lack of human resources for nursing and unsound social medical insurance systems, many patients with femoral neck fractures need to be cared for at home. Therefore, the burden of helping patients recover mainly falls on the shoulder of family caregivers, who become the family caregivers of such patients. The concept of a family caregiver refers to a person who has a strong emotional bond with the patient, is a family member, and can provide emotional support and nursing support to the patient during the illness of a family member [6]. Family caregivers play a key role in the management of patients with femoral neck fracture, as they take on the physical, emotional, medical, and financial responsibilities of caring for their sick relatives and thereby experience tremendous pressure and caregiver burden [7, 8].

Many factors, including disease-related factors, sociopsychological factors, and clinical and sociodemographic factors, are related to the burden of caregivers [9]. Disease-related variables include the patient’s functional status, cognitive function, pain score, and Harris score [10–13]. Clinical and sociodemographic factors include variables related to patients and caregivers. Patient-related variables include age, gender, and education level. Caregiver-related variables include age, gender, education level, place of residence, relationship with patient, monthly income, and length of care [10, 11, 13]. Socio-psychological factors encompass social support and self-efficacy [10, 13, 14]. In China, little literature is available on factors related to the burden of caregivers for patients with femoral neck fracture. Concerning the burden of caregivers, understanding specific factors affecting the burden of caregivers, and satisfying the needs of caregivers can, on the one hand, lessen the pressure on caregivers, enhance their care and nursing skills, and speed up patient recovery; on the other hand, they can support medical and health institutions and the government to improve the current situation of care for patients with femoral neck fracture. Therefore, this study analyzed and evaluated the clinical data of patients and their family caregivers, combined with related factors affecting the caregiver burden in the relevant literature, for the purpose of clarifying the factors that affect the burden of family caregivers for elderly patients with femoral neck fracture.

## Methods

### Study participants

We investigated 183 family caregivers of elderly patients aged ⩾ 65 years undergoing HA for a femoral neck fracture. A cross-sectional study was conducted. We employed non-probability sampling methods to select participants. We included any family members aged between 18 and 75 who had lived with and cared for an elderly patient with a femoral neck fracture for longer than 3 months, who were able to speak, read, and understand Chinese, and who were willing to participate in the study. We excluded those who suffered from a mental disorder; those with communication difficulties or difficulty understanding the survey questions; those who had cognitive dysfunction; those who did not sign a written informed consent form; those who had cared for patients for less than 3 months; and those who declined to participate in the study or withdrew halfway. The ethics review committee of the Shaanxi Provincial People’s Hospital approved the study protocol. All the patients and caregivers provided written informed consent for the use and publication of data for research purposes.

### Data collection

The data were collected from Shaanxi Provincial People’s Hospital by trained personnel, under the direction of the first author, between January 2016 and June 2019. We conducted a sociodemographic questionnaire of patients with femoral neck fracture and their family caregivers. The demographic data collected from patients included age, gender and education level. The demographic data collected from family caregivers included age, gender, education level, place of residence, relationship with patient, duration of care, and the ratio of medical expenses to monthly income per capita. We obtained the patients’ clinical data from their medical records, such as the fracture site, functional status, pain level, and Harris score. After the researcher explained the study’s objectives, methods, benefits, and potential risks to family caregivers, those who agreed to participate in the study signed a written informed consent form. Subsequently, we conducted a face-to-face interview with them.

### Evaluation standard

The burden of family caregivers was assessed using the Zarit Burden Interview (ZBI) [15]. The Chinese version of the ZBI was reliable and valid for Chinese caregivers [16]. The total score of care burden can be divided into 3 levels: 0–20 indicates little or no burden; 21–39 indicates a medium burden; and 40–88 indicates a severe burden. The scale’s overall internal consistency is very high (Cronbach’s alpha = 0.88) [13].

Social support is measured using the Social Support Rating Scale (SSRS) [17], which consists of 3 subscales with 10 items. A higher score indicates a higher level of social support. The scale’s overall internal consistency is very high (Cronbach’s alpha = 0.90) [13].

The self-efficacy of caregivers was measured using the General Self-Efficacy Scale (GSE) [18]. A higher score indicates a higher self-efficacy. The scale’s overall internal consistency is very high (Cronbach’s alpha = 0.91) [13].

### Statistical analysis

The data were processed using SPSS 19.0 statistical software (SPSS, IL, USA). Enumeration data were expressed as percentages (%), and measurement data were expressed as “*x* ± s”. Spearman’s rank correlation coefficient was used to calculate the correlation. Single variables and important factors in the correlation analysis were entered into a stepwise multiple linear regression, in which the caregiver burden was used as a dependent variable. *P* < 0.05 was considered statistically significant.

## Results

The study recruited 183 caregivers in total, 2 of whom refused to participate and 5 of whom quit halfway. Ultimately, the sample size (with a response rate of 96.2%) was 176.

### Characteristics of the patients and caregivers

A total of 176 patients with femoral neck fracture ranged from 21 to 79 years of age (with a mean 68.15 ± 6.37 years), and 54.0% were female. A total of 63.1% of the patients had an educational background of primary school or below, 12.5% of the patients were dependent on daily activities, 44.3% had moderate or severe pain, 80.7% had a Harris score greater than 70, and 27.2% had a bilateral femoral neck fracture (Table 1).

**Table 1:** Demographic and clinical characteristics of patients and their primary caregivers

Variables	Patients (*n* = 176)	Caregivers (*n* = 176)
Age (years old)	68.15 ± 6.37	69.28 ± 7.19
Gender		
Female	95 (54.0%)	84 (47.7%)
Male	81 (46.0%)	92 (52.3%)
Educational level		
≦Primary school	111 (63.1%)	118 (67.0%)
≧Secondary school	65 (36.9%)	58 (33.0%)
Place of residence		
Countryside	N/A	74 (42.0%)
City	N/A	102 (58.0%)
Relationship		
Spouse	N/A	148 (84.1%)
Other	N/A	28 (15.9%)
Care time		
<3 months	N/A	114 (64.8%)
> 3 months	N/A	62 (35.2%)
The ratio of medical expenses and monthly income per capita		
< 10/1	N/A	28 (15.9%)
10/1–5/1	N/A	105 (59.7%)
> 5/1	N/A	43 (24.4%)
Fracture site		
Left side	61 (34.7%)	N/A
Right side	67 (38.1%)	N/A
Both sides	48 (27.2%)	N/A
Functional status (activities of daily living) [12]		
Fully independent	65 (36.9%)	N/A
Fully dependent	22 (12.5%)	N/A
Other	89 (50.6%)	N/A
Harris score [13]		
≧ 90	102 (58.0%)	N/A
70–89	40 (22.7%)	N/A
*<* 70	34 (19.3%)	N/A
Pain level, VAS score		
≦ 3	98 (55.7%)	N/A
4–6	51 (29.0%)	N/A
≧ 7	27 (15.3%)	N/A

Values are presented as the mean and standard deviation or as the number and percentage.

*VAS* visual analog scale

A total of 176 caregivers were all from the patients’ families, ranging from 21 to 79 years of age (with a mean of 69.28 ± 7.19 years). A total of 67.0% of them had an education of primary school or below, and most of them lived in urban areas (58%). A total of 84.1% of the caregivers were patients’ spouses. A total of 35.2% of caregivers took care of patients for more than 3 months, and 75.6% of them had a ratio of medical expenses to monthly income per capita of less than 5/1 (Table 1).

### Correlations of caregiver burden with the SSRS and GSE scores

The average ZBI score of family caregivers was 37.8 ± 8.9. Among them, 17.3% of the caregivers had a ZBI rating of little or no burden, 62.9% had a rating of moderate burden, and 19.8% had a severe burden. The average SSRS score of family caregivers was 41.9 ± 7.2, and the average GSE score was 26.1 ± 6.9. This study analyzed the correlations between ZBI, SSRS, and GSE scores to assess the relationship between caregiver burden and self-efficacy and social skills. The analysis results showed a negative correlation between ZBI scores and SSRS and GSE scores.

### Factors associated with caregiver burden

#### Univariate analysis

The univariate analysis showed that the care burden was not related to the patient’s age, gender, education level, fracture site, or other characteristics but rather was significantly related to the patient’s functional status, Harris score, and pain level (Table 2).

**Table 2:** Univariate analysis of caregiver burden and patient variables

Variable	Little/no burden	Medium burden	Serious burden	Statistical significance
Age (years old)	67.23 ± 8.13	70.27 ± 7.83	69.57 ± 7.86	*P* > 0.05
Gender				
Female	17 (56.7%)	61 (55.0%)	17 (48.6%)	*P* > 0.05
Male	13 (43.3%)	50 (45.0%)	18 (51.4%)	*P* > 0.05
Educational level				
≦Primary school	20 (66.7%)	69 (62.2%)	22 (62.9%)	*P* > 0.05
≧Secondary school	10 (33.3%)	42 (37.8%)	13 (37.1%)	*P* > 0.05
Fracture site				
Bilateral	8 (26.7%)	30 (27.0%)	10 (28.6%)	*P* > 0.05
Other	22 (73.3%)	81 (73.0%)	25 (71.4%)	*P* > 0.05
Functional status (activities of daily living)				
Fully independent	11 (36.7%)	41 (36.9%)	13 (37.1%)	*P <* 0.05
Other	19 (63.3%)	70 (63.1%)	22 (62.9%)	*P <* 0.05
Harris score [14]				
≧ 70	24 (80.0%)	90 (81.1%)	28 (80.0%)	*P <* 0.05
*<* 70	6 (20.0%)	21 (18.9%)	7 (20.0%)	*P <* 0.05
Pain level, VAS score				
≦ 3	17 (56.7%)	62 (55.9%)	19 (54.3%)	*P <* 0.05
> 4	13 (43.3%)	49 (44.1%)	16 (45.7%)	*P <* 0.05

The univariate analysis indicated that caregiver burden was not associated with the caregiver’s age, gender, education level, relationship with the patient, and other characteristics but rather was significantly correlated with the caregiver’s place of residence, care duration, and the ratio of medical expenses to monthly income per capita (Table 3).

**Table 3:** Univariate analysis of caregiver burden and caregiver variables

Variable	Little/no burden	Medium burden	Severe burden	Statistical significance
Age (years old)	70.12 ± 6.45	71.45 ± 4.01	68.10 ± 7.65	*P* > 0.05
Gender				
Female	14 (46.7%)	53 (47.7%)	17 (48.6%)	*P* > 0.05
Male	16 (53.3%)	58 (52.3%)	18 (51.4%)	*P* > 0.05
Educational level				
≦Primary school	21 (70.0%)	74 (66.6%)	23 (65.7%)	*P* > 0.05
≧Secondary school	9 (30.0%)	37 (33.3%)	12 (34.3%)	*P* > 0.05
Place of residence				
Countryside	12 (40.0%)	47 (60.3%)	15 (61.5%)	*P* < 0.05
City	18 (60.0%)	64 (39.7%)	20 (38.5%)	*P* < 0.05
Relationship				
Spouse	25 (83.3%)	94 (84.7%)	29 (82.9%)	*P* > 0.05
Other	5 (16.7%)	17 (15.3%)	6 (17.1%)	*P* > 0.05
Care duration				
*<*9 months	19 (63.3%)	72 (64.9%)	23 (65.7%)	*P* < 0.05
> 9 months	11 (36.7%)	39 (35.1%)	12 (34.3%)	*P* < 0.05
The ratio of medical expenses to monthly income per capita				
≦ 5/1	23 (76.7%)	84 (75.7%)	26 (74.3%)	*P* < 0.05
> 5/1	7 (23.3%)	27 (24.3%)	9 (25.7%)	*P* < 0.05

#### Multivariate analysis

The multivariate analysis revealed that caregiver burden was positively correlated with the patient’s functional status, Harris score, pain score, GSE score, SSRS score, and the ratio of medical expenses to monthly income per capita. These identified factors could explain approximately 60% of the variation in caregiver burden.

## Discussion

HA is conducive to improving joint function recovery and reducing complications for patients with femoral neck fracture, and it has been widely applied clinically as a surgical method to treat elderly patients with femoral neck fracture. This study analyzed the clinical data of elderly patients with femoral neck fracture and that of their family caregivers. Combined with reported factors that affect the caregiver burden, this study analyzed the burden-related factors of caregivers of patients who underwent an operation after femoral neck fracture and primarily revealed the burden borne by caregivers who cared for patients with femoral neck fracture in this area of China. This study showed that most of the family caregivers of patients with femoral neck fracture came under considerable caregiver burden, and the important factors that were obviously correlated with the care burden were the patient’s functional status, Harris score, pain score, GSE score, SSRS score, and the ratio of medical expenses to monthly income per capita.

In this study, the sociodemographic characteristics of caregivers indicated that family caregivers were the patients’ spouses, who had a low education level and were advanced in age. However, these demographic characteristics did not aggravate the caregiver burden of caregivers. This is consistent with the results of related parallel studies [10], which may be due to the similar sociodemographic characteristics of caregivers in Western China as well as the consistency in cultural or behavioral factors. This study showed that the patient’s functional status was positively correlated with the caregiver burden. Patients with femoral neck fracture need increased care and personal nursing in their daily lives due to the partial or total loss of self-care ability after the operation, thereby increasing the care burden. This study also showed that the degree of pain that patients experienced was positively correlated with the burden of care. Severe pain affected the quality of life of patients and was injurious to their body and mind. In addition, pain requires continuous analgesic therapy as well as sustained emotional support, which not only increases the caregiver’s expenditure but also contributes to mental health problems in the caregiver and ultimately adds to the burden of care. In this study, most of the family caregivers had a ratio of medical expenses to monthly income per capita of less than 5/1. As a result, these caregivers experienced enormous life pressures, heightening the burden of caregivers. In summary, caregivers had to pay medical expenses and support the family while caring for patients. They were under tremendous pressure physically, psychologically, and economically. The persistent stress load affected the health of caregivers, and the health status of caregivers also directly influenced patients’ rehabilitation and quality of life. Therefore, it is necessary for health care institutions and government to introduce specific measures and policies to reduce the care burden for the families of elderly caregivers, such as hospital strengthening care skills training. Additionally, through refining the rehabilitation training guidance and increasing emotional support, the government increases the provision and support for the aged, thus increasing financial support and reducing medical expenses.

Previous studies have demonstrated that caregivers’ social support and self-efficacy are negatively correlated with caregiver burden [17, 18]. Caregivers feel that social support affects their anxiety and depression levels, as it can effectively lessen the severity of their stress [19]. However, when the targeted support provided by professionals to caregivers is not satisfactory, the pressure felt by caregivers augments [20]. This study shows that social support can lessen caregiver burden, consistent with the above studies. In China, due to the influence of traditional culture and a lack of available services (for example, in single-child families, offspring cannot take care of elderly parents due to work reasons), caregivers often do not receive substantial support from other members of the family, while social support from the family and professional institutions makes it easier for family caregivers to take care of patients and themselves, cope with stress, and minimize the caregiver burden [21, 22]. Therefore, to reduce the burden of caregivers, professional medical and health institutions can take measures to improve the current situation, provide appropriate social support to help caregivers, and reduce their burden. Self-efficacy is the individual’s confidence or belief in a new organization and its executive capability in a particular field. Individuals with high self-efficacy will carry out the task more proactively, while individuals with low self-efficacy may evade it due to a sense of inability and helplessness [23]. Self-efficacy can bring about more positive emotions and contribute to health [24], thereby reducing the caregiver burden [25]. The results of the questionnaire in this study reveal that self-efficacy is negatively correlated with caregiver burden, consistent with the above results, possibly because family caregivers with high self-efficacy have strong self-regulation abilities, are psychologically healthier, and can better cope with care pressure [26]. Specifically, individuals with a strong ability in emotion regulation can adjust their emotions to fit in with the situation they are in at the moment. Generally, they have more positive emotional experiences, which can in turn promote their mental health and enhance their happiness, make them more flexible in thought and encourage them to come up with solutions to the problem. Given our research findings, more intervention measures should be taken to improve the self-efficacy of family caregivers. Intervention measures can include professional trainings or psychological support, which may help reduce the burden of family caregivers of elderly patients with femoral neck fracture.

This study also has some limitations, as it only involves an orthopedic center in Western China. Considering the cross-sectional nature of this study, it is not plausible to establish causality between the results and the survey variables. To better understand the factors influencing the caregiver burden among family caregivers of patients with femoral neck fracture in China, it is also necessary to conduct a longitudinal cohort study to gain deeper insight into the factors involved in the care burden of family caregivers and the impact of these factors on caregivers. In addition, the inclusion of non-randomly selected participants may lead to unpredictable biases. Despite the above limitations, this study has identified factors closely related to the caregiver burden and has proposed improvement strategies and intervening measures, which will eventually contribute to better patient care.

## Conclusion

This study focuses on the caregiver burden among family caregivers of patients with femoral neck fracture. Family caregivers of patients with femoral neck fracture usually have a poor quality of life and experience tremendous psychological and economic pressure. Most of them are subject to a heavy burden. The burden of the caregiver is directly related to the patient’s functional status, pain score, Harris score, GSE score, SSRS score, and the ratio of medical expenses to monthly income per capita, while it is negatively correlated with social support and self-efficacy. Patients with femoral neck fracture should be given more attention. Meanwhile, social support should be provided to family caregivers, and efforts should be made to improve their self-efficacy.

## Data Availability

The datasets supporting the conclusions of this article are included within the article. The raw data can be requested from the corresponding author on reasonable request.

## Abbreviations

SSRS: Social Support Rating Scale
ZBI: Zarit Burden Interview
GSE: General Self-Efficacy Scale
SD: Standard deviation
